# Effects of Salt Stress on the Antioxidant Activity and Malondialdehyde, Solution Protein, Proline, and Chlorophyll Contents of Three *Malus* Species

**DOI:** 10.3390/life12111929

**Published:** 2022-11-18

**Authors:** Dajiang Wang, Yuan Gao, Simiao Sun, Xiang Lu, Qingshan Li, Lianwen Li, Kun Wang, Jihong Liu

**Affiliations:** 1 Xinjiang Production and Construction Corps Key Laboratory of Special Fruits and Vegetables Cultivation Physiology and Germplasm Resources Utilization, Agricultural College of Shihezi University, Shihezi 832003, China; 2 National Repository of Apple Germplasm Resources, Key Laboratory of Horticulture Crops Germplasm Resources Utilization, Ministry of Agriculture and Rural Affairs of the People’s Republic of China, Research Institute of Pomology, Chinese Academy of Agricultural Sciences (CAAS), Xingcheng 125100, China; 3 Key Laboratory of Horticultural Plant Biology (MOE), College of Horticulture and Forestry Sciences, Huazhong Agricultural University, Wuhan 430070, China

**Keywords:** *Malus* seedlings, NaCl treatments, enzyme activity, membrane damage, osmotic regulation

## Abstract

Understanding the different physiological responses of *Malus* species under salt stress in the seedling stages will be useful in breeding salt-tolerant dwarfing apple rootstocks. Seedlings of *Malus Zumi* (Mats.) Rehd. (*M. zumi*), *Malus sieversii* (Led.) Roem. (*M. sieversii*), and *Malus baccata* (L.) Borkh. (*M. baccata*) were treated with solution of 0, 0.20%, 0.40%, and 0.60% salinity. Physiological parameters of their leaves and roots were measured at 0 d, 4 d, 8 d and 12 d after salinity treatments. Superoxide dismutase (SOD), peroxidase (POD), catalase (CAT), malondialdehyde (MDA), solution protein (SP), and proline (PRO) initially increased and then decreased. The activities and contents of these parameters were higher in the 0.40% and 0.60% NaCl treatments than in the 0.20% treatment and in the 0% control. *M. zumi* was the most resistant to salt stress, showing the lowest content of MDA in the leaves and roots, which increased slightly under salt stress. *M. baccata* had the highest increase in both the content and proportion of MDA. High enzyme activity was shown to play an important role in the salt resistance of *M. zumi*. Moreover, it can be speculated that there are other substances that also play a major role. We found that osmotic regulation played a key role in response to salt stress for *M. baccata* even though it was sensitive to salt stress. For *M. sieversii*, both the osmotic regulation and enzymatic antioxidants were observed to play a major role in mitigating salt stress.

## 1. Introduction

More than 800 million hectares of land and 32 million hectares of agricultural land are affected by salinity stress globally [1,2]. Moreover, it is estimated that soil salinization will cause deterioration of 50% of the land by the year 2050 [3]. Under salt stress, almost all plants exhibit adverse effects [4]. Salt stress causes water loss, iron ion absorption inhibition in roots, a reduction in the photosynthetic efficiency of leaves, and diminished tree growth, all of which seriously affect the healthy growth and yield formation of plants, including apple trees [5,6,7]. A salty environment produces two kinds of stress factors in plants: osmotic stress and ionic toxicity. The former obstructs water absorption in plants; the latter is toxic to the physiological function of plant metabolism. Moreover, both can lead to the production of reactive oxygen species (ROS), which damage the structure of cell membranes [8]. The changes in POD, SOD, and CAT activities can reflect the ability to scavenge ROS under stress in plants. SOD can dismutate O^2−^ to O_2_ or H_2_O_2_, CAT can catalyze H_2_O_2_ to H_2_O and O_2_, and POD can direct oxidation of phenol or amine compounds with H_2_O_2_ as electron acceptor to eliminate the toxic H_2_O_2_ and phenol amine [9,10]. SP and PRO contents reflect the ability to overcome osmotic pressure. Plants synthesize PRO, SP, soluble sugars, and other osmolytes to promote osmotic balance at the cellular level; the biosynthesis of PRO is activated by stress [10,11]. MDA content, one of the most important products of membrane lipid peroxidation, reflects the degree of damage to the membrane system under biotic and abiotic stresses [12,13,14,15,16]. Salt stress was shown to cause lipid peroxidation as well as the accumulation of soluble sugars and PRO, and to increase the activity of antioxidant enzymes in both salt-resistant and salt-sensitive bread wheat [17]. Increasing NaCl was shown to increase the SOD and POD activities, as well as the PRO and MDA contents, in linseed [18]. MDA content and SOD, CAT, and POD activities were shown to increase with increasing salinity in lentils [19]. The enhancing and transporting of PRO in plant organs are important survival strategies against salt stress; exogenous PRO may enhance resistance to salt stress in lupine [20,21].

Apple, one of the most popular fruits globally, plays a major role in poverty alleviation and rural revitalization in north and northwest China. At present, the apple dwarfing rootstocks widely used in China and abroad—M26, MM106, M9, etc.—are not tolerant to salt [22]. The soil salt content in certain regions in north and northwest China exceeds 0.4% [23,24,25]. Northwest China is the new dominant producing area of apple. Salinity stress has, to a certain extent, restricted the development of the apple industry in these areas. Using salt land to grow apples is one of the ways to expand the apple industry in Northwest China. The breeding of salt-tolerant apple rootstock is an important guarantee to achieve this approach and is the theoretical basis for salt-tolerant breeding to understand the physiological mechanism of salt-tolerant *Malus* species.

There are approximately 55 species of *Malus* around the world [26]. Different species have been developed with special characteristics to adapt to the natural environment in the distribution center after a long period of natural selection. For example, *Malus xiaojinensis* Cheng et Jiang., *Malus toringoides* (Rehd.) Hughes., and *Malus kansuensis* (Batal.) Schneid. are tolerant to drought; *Malus hupehensis* (Pamp.) Rehd. and *Malus toringoides* (Rehd.) Hughes. are tolerant to waterlogging; *M. baccata* and *M. sieversii* are tolerant to cold; *Malus robusta* (Carr.) Rehd., *M. sieversii*, and *Malus sikkimensis* (Wenzig.) Koehne. are tolerant to salt; and even *M*. *zumi* is tolerant to a 0.60% salt content in soil [27,28]. There are three ways to avoid ionic toxicity in plants: salinity dilution, salinity regionalization, and salt rejection. Overcoming osmotic stress mainly depends on the content of osmotic regulating substances [29,30]. For *Malus* plants, the main mechanisms of salt stress resistance are salt rejection and ion regionalization. However, they have a long history of heredity and evolution, with different species exhibiting different tolerances to salt stress, and thus the adaptation mechanisms to salt stress are not all the same. Whether different *Malus* species take advantage of the same substance to scavenge ROS or regulate osmotic stress remains unknown. Therefore, understanding the physiological basis of salt resistance in different *Malus* species is very important for breeding salt-tolerant rootstocks.

Plants may be more tolerant to salt in the seedling stage than in the other growth stages [31]. *M. zumi*, *M. sieversii*, and *M. baccata* are high-resistance, medium-resistance, and salt-sensitive, respectively. The latter two are widely used as rootstocks in northwest and northeast China, but how they physiologically differ in terms of salt stress resistance remains unclear.

In the present study, the activities of POD, SOD, and CAT and the contents of MDA, SP, PRO, CHLa, and CHLb were compared in the three species under different NaCl treatments during the seedling period. We aimed to evaluate the effects on the physiological parameters of salt stress to elucidate the adaptive mechanisms of different *Malus* species to salinity stress. Our findings can be used as the basis for the breeding of salt-tolerant dwarfing rootstocks.

## 2. Materials and Methods

### 2.1. Plant Materials

*M. zumi*, *M. sieversii*, and *M. baccata* trees were planted in the National Repository of Apple Germplasm Resources (Xingcheng, Liaoning, China) in 2007. *M. zumi*, *M. sieversii*, and *M. baccata* are genotypes that are characterized as high-resistance, medium-resistance and salt-sensitive, respectively. The seeds of *M. zumi*, *M. sieversii*, and *M. baccata* were collected in the autumn of 2019 and were laminated for 60 days at 4 °C starting in late January of 2020. After germination, seeds were sown in a seedling tray in April and transplanted into plastic pots in June. One seedling was planted per pot.

### 2.2. Experimental Design

All experiments took place in a greenhouse. During the experiments, the average temperature was approximately 28 °C. The lowest temperature was approximately 16 °C and the highest temperature was 33 °C. The relative air humidity was 50–60%. A total of 200 seedlings that exhibited uniform growth, were 1 year old, and were approximately 30 cm tall were selected from each species, with 50 seedlings per group. Four groups were irrigated with either 0, 0.20%, 0.40%, or 0.60% NaCl solution, respectively, three times from 10 to 17 July 2020. Samples of roots and leaves were collected at 0, 4, 8, and 12 days after the last round of irrigation for each group. Leaves or roots of three plants were mixed for each group as one replicate with three replicates per group. The samples were rinsed with tap water to remove the soil and other surface debris and then washed with distilled water. All the samples were frozen in liquid nitrogen and stored at −80 °C.

### 2.3. Physiological Parameter Measurements

The nitroblue tetrazolium (NBT) method was used to determine the SOD activity [32]. The superoxide anion (O^2−^) is produced by the xanthine and xanthine oxidase reaction system. O^2−^ reduces nitroblue tetrazole to generate blue formazan, for which the maximum absorption peak is 560 nm. SOD scavenges O^2−^, which results in formazan being inhibited. The more blue the reaction liquid, the lower the SOD activity. The experimental steps used were those described in the kit instructions (kit series no.: SOD-2-Y, Comin Biotechnology, Suzhou, China; www.cominbio.com, accessed on 15 May 2020). A total of 1 mL of blank tube solution and measuring tube solution was absorbed into a glass colorimetric dish, and the absorbance value at 560 nm was recorded as Ab560 for the blank tube and Am560 for the measuring tube.

$$\mathrm{PI}\;\left(\mathrm{percentage}\;\mathrm{of}\;\mathrm{inhibition}\right)=\frac{\mathrm{Ab}560-\mathrm{Am}560}{\mathrm{Ab}560}\times 100\%$$

$$\mathrm{SOD}\;\mathrm{activity}\;\left(U/g,\;\mathrm{FW}\right)=11.4\times \frac{\mathrm{PI}}{0.1\times \left(1-\mathrm{PI}\right)}$$

The guaiacol method was used to determine the POD activity [33]. POD catalyzes the oxidation of specific substrates with H_2_O_2_ and has a characteristic light absorption at 470 nm. The experimental steps used were those described in the kit instructions (kit series no.: POD-2-Y, Comin Biotechnology, Suzhou, China; www.cominbio.com, accessed on 15 May 2020). A total of 1 mL of supernatant was added into a glass colorimetric dish; the absorbance value at 470 nm was recorded as Ab470, and the value 1 min later was recorded as Al470.

$$\mathrm{POD}\;\mathrm{activity}\;\left(U/g,\;\mathrm{FW}\right)=\frac{2000\times \left(\mathrm{Al}470-\mathrm{Ab}470\right)}{0.1}$$

The ultraviolet absorption method was utilized to determine the CAT activity [34]. H_2_O_2_ has a characteristic absorption peak at 240 nm, and CAT can decompose H_2_O_2_, so the absorbance of the reaction solution at 240 nm decreased with the reaction time. CAT activity could then be calculated according to the change rate of the absorbance. The experimental steps used were those described in the kit instructions (kit series no.: CAT-2-Y, Comin Biotechnology, Suzhou, China; www.cominbio.com, accessed on 17 May 2020). The absorbance value at 240 nm was recorded as Ab240, and the value 1min later was recorded as Al240.

$$\mathrm{CAT}\;\mathrm{activity}\;\left(U/g,\;\mathrm{FW}\right)=\frac{687\times \left(\mathrm{Ab}240-\mathrm{Al}240\right)}{0.1}$$

The thiobarbituric acid (TBA) method was applied to measure the MDA content. MDA combined with thiobarbituric acid (TBA) to produce a red product with a maximum absorption peak at 532 nm. The content of lipid peroxide in the sample could be estimated after colorimetry; the MDA content was calculated as the difference between the absorbance values at 532 and 600 nm. The experimental steps used were those described in the kit instructions (kit series no.: MDA-2-Y, Comin Biotechnology, Suzhou, China; www.cominbio.com, accessed on 15 May 2020). A total of 1 mL of upper solution was absorbed into a glass colorimetric dish; the absorbance values at 532 and 600 nm were recorded as A532 and A600, respectively.

$$\mathrm{MDA}\;\mathrm{content}\;\left(\mathrm{nmol}/g,\;\mathrm{FW}\right)=\frac{25.8\times \left(A532-A600\right)}{0.1}$$

The bicinchoninc acid (BCA) method was performed to determine the SP content. Under alkaline conditions, cysteine, tryptophan, tyrosine, and peptide bonds in proteins can reduce Cu^2+^ to Cu^+^. Two molecules of BCA combined with Cu^+^ to form a purple complex, which had an absorption peak at 562 nm. The experimental steps used were those described in the kit instructions (kit series no.: BCAP-2-W, Comin Biotechnology, Suzhou, China; www.cominbio.com, accessed on 15 May 2020). The absorbance values of a blank tube, standard tube, and measuring tube at 562 nm was recorded as Ab562 for the blank tube, As562 for the standard tube, and Am562 for measuring tube.

$$\mathrm{SP}\;\mathrm{content}\;\left(\mathrm{mg}/g,\;\mathrm{FW}\right)=\frac{0.5\times \left(\mathrm{Am}562-\mathrm{Ab}562\right)}{0.1\times \left(\mathrm{As}562-\mathrm{Ab}562\right)}$$

The sulfosalicylic acid (SSA) method was used to determine the PRO content. PRO was extracted with sulfosalicylic acid and reacted with an acidic ninhydrin solution to produce a red color after heating. The absorbance value was measured at 520 nm after extraction with methylbenzene. The experimental steps were those described in the kit instructions (kit series no.: PRO-2-Y, Comin Biotechnology, Suzhou, China; www.cominbio.com, accessed on 15 May 2020). The absorbance value was recorded as A520 at 520 nm.

$$\mathrm{PRO}\;\mathrm{content}\;\left(µg/g,\;\mathrm{FW}\right)=\frac{19.2\times \left(A520+0.0021\right)}{0.1}$$

Determination of the CHLa and CHLb content was performed according to the experimental steps described in the kit instructions (kit series no.: CPL-2-G, Comin Biotechnology, Suzhou, China; www.cominbio.com, accessed on 15 May 2020). The absorbance values at 663 and 645 nm were determined and were denoted as A663 and A645, respectively.

$$\mathrm{CHLa}\;\mathrm{content}\;\left(\mathrm{mg}/g,\;\mathrm{FW}\right)=\frac{\left(12.7\times A663-2.69\times A645\right)\times \mathrm{Ve}\times D}{m\times 1000}$$

$$\mathrm{CHLb}\;\mathrm{content}\;\left(\mathrm{mg}/g,\;\mathrm{FW}\right)=\frac{\left(22.9\times A663-4.68\times A645\right)\times \mathrm{Ve}\times D}{m\times 1000}$$

where Ve is the extraction volume, D is the multiple dilution, and m is the sample weight.

### 2.4. Statistical Analysis

The experimental data collection and analysis were performed using Microsoft Excel 2010. The physiological parameters were subjected to an analysis of variance (ANOVA) using SPSS 19. The significant difference level, which was calculated using Tukey’s test, was used to compare the differences among treatments and the control. The PCA was conducted using Origin 2019b software.

## 3. Results

### 3.1. Dynamic Effects on Physiological Parameters of Leaves of Malus Plants under Salinity Stress

**Figure 1:**
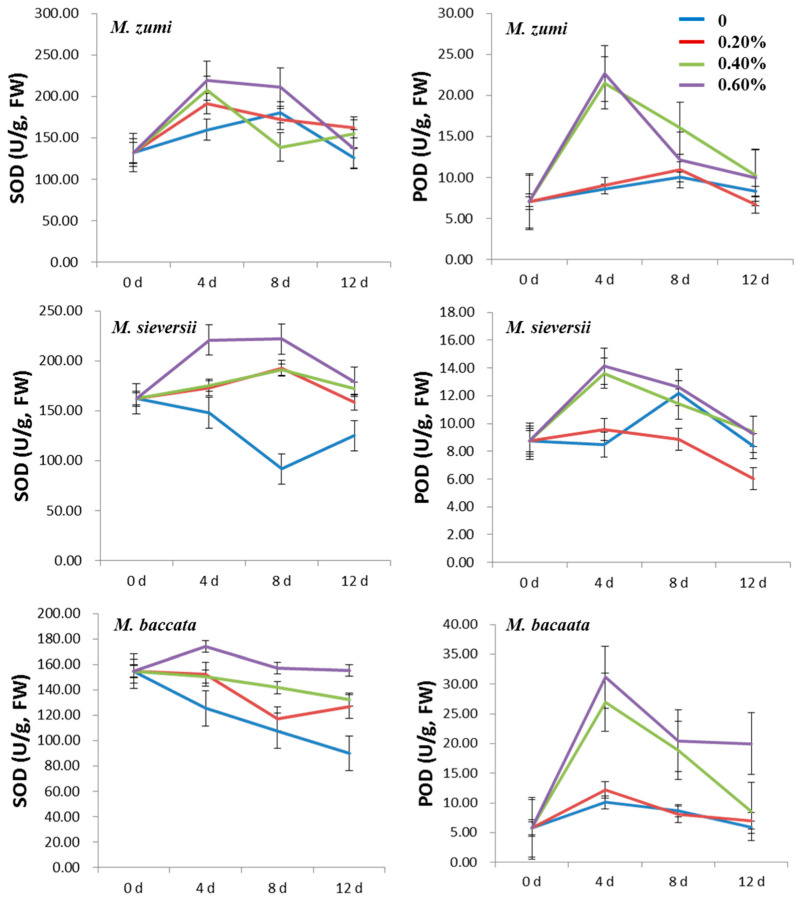
Change curve of leaf SOD and POD activities for the three *Malus* species under four levels of NaCl treatments.

**Figure 2:**
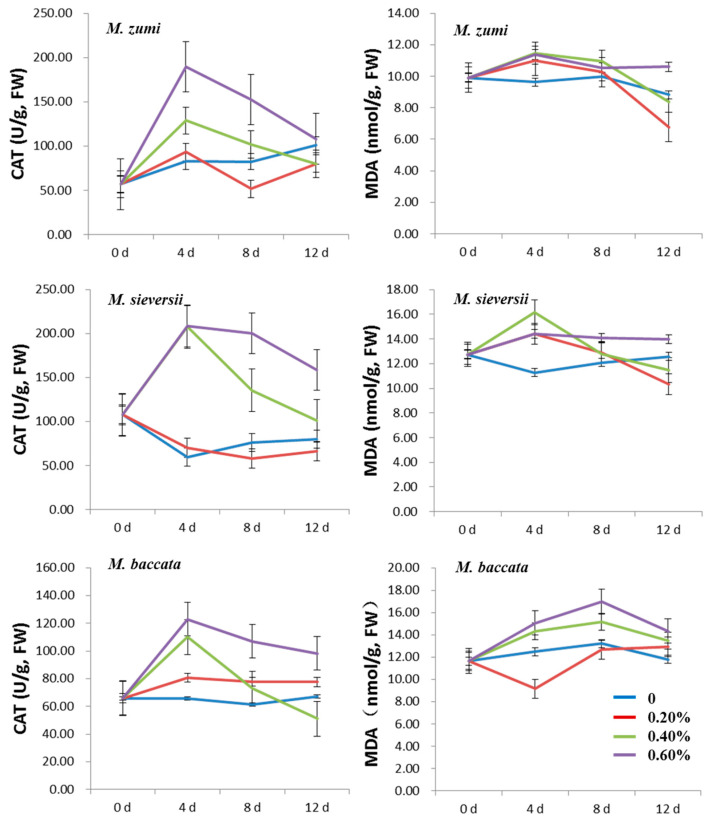
Change curve of leaf CAT activity and MDA content for three *Malus* species under four levels of NaCl treatments.

The SOD, POD, and CAT activities of the three *Malus* species increased at first and then decreased in the leaves in the 0.20%, 0.40% and 0.60% NaCl treatment groups (Figure 1 and Figure 2; Table S1). The activities of SOD, POD, and CAT were higher in the 0.40% and 0.60% NaCl treatments groups than in the 0.20% NaCl treatments group and in the 0% control. For all treatments, the highest activity of SOD, POD, and CAT was exhibited on the fourth day after NaCl treatment. At the peak time, the SOD activity increased by 49.33%, 38.54%, and 37.11% for *M. sieversii*, *M. baccata*, and *M. zumi*, respectively, in the 0.60% NaCl treatment group compared with the control treatment; moreover, the POD activity increased by 66.63%, 208.01%, and 164.22%, respectively; and the CAT activity increased by 250.68%, 86.67%, and 128.73%, respectively. POD increased the most for *M. baccata*, while SOD and CAT increased the most for *M. sieversii.* The increase in the SOD, POD, and CAT activities fell in the midrange for *M. zumi*. After 12 days of treatment, there was little difference between treatments and the control in terms of SOD, POD, and CAT activities in *M. zumi* and POD activity in *M. sieversii*, but the SOD, POD, and CAT activities remained obviously higher in *M. baccata* than in the control.

For all the NaCl treatments, the MDA content increased at first and then decreased in the three *Malus* species (Figure 2; Table S1). The MDA content was higher in the 0.40% and 0.60% NaCl treatments groups than in the 0.20% NaCl treatments group and in the control except for *M. zumi*. The times of peak occurrence were different, reaching a peak on the fourth day after NaCl treatment for *M. zumi* and *M. sieversii* and on the eighth day after NaCl treatment for *M. baccata*. At the time of peak MDA content for the 0.60% NaCl treatment group, the MDA content in *M. sieversii*, *M. baccata*, and *M. zumi* increased by 27.66%, 28.64%, and 18.17%, respectively, compared with the control treatment. After 12 days of treatment, there was little difference between treatments and the control for the three species, but *M. zumi* had an overall lower MDA content than the other two species both in the treatments and in the control.

**Figure 3:**
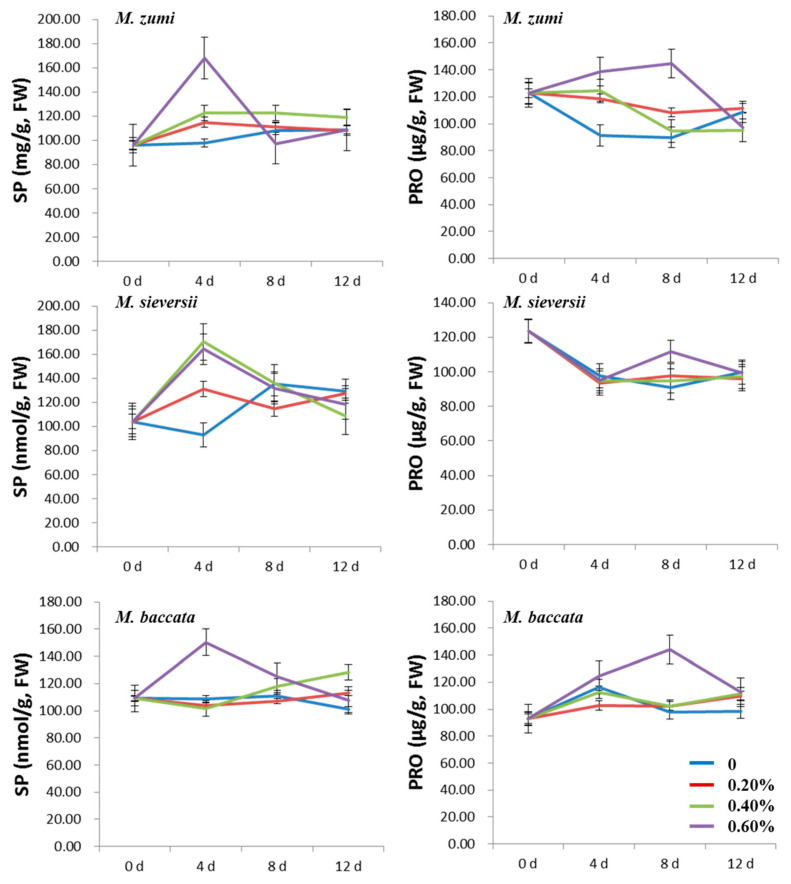
Change curve of leaf SP and PRO contents for the three *Malus* species under four levels of NaCl treatments.

The contents of SP and PRO increased at first and then decreased in all NaCl treatments for the three *Malus* species (Figure 3; Table S1). The SP and PRO contents were higher in the 0.60% NaCl treatment group than those in the 0.20% and 0.40% NaCl treatment groups and the control, with the exception of the PRO content in *M. sieversii*. The peak time was different for SP and PRO, with the former peaking on the fourth day after NaCl treatments and the latter on the eighth day after NaCl treatments. With the increasing NaCl concentration, the SP and PRO contents in the 0.20% NaCl treatment group and the control for the three *Malus* species was lower than in the 0.40% and 0.60% groups. Compared with the control treatment, in *M. sieversii*, *M. baccata*, and *M. zumi* subjected to 0.60% NaCl treatments, the content of SP increased by 76.58%, 38.26%, and 71.68%, respectively; and the content of PRO increased by 22.95%, 47.45%, and 61.07%, respectively, when considering their values at peak. After 12 days of treatment, there was little difference between treatments and the control for SP and PRO in the three species except for the SP content in *M. baccata*, which was slightly higher in the treatments than in the control.

**Figure 4:**
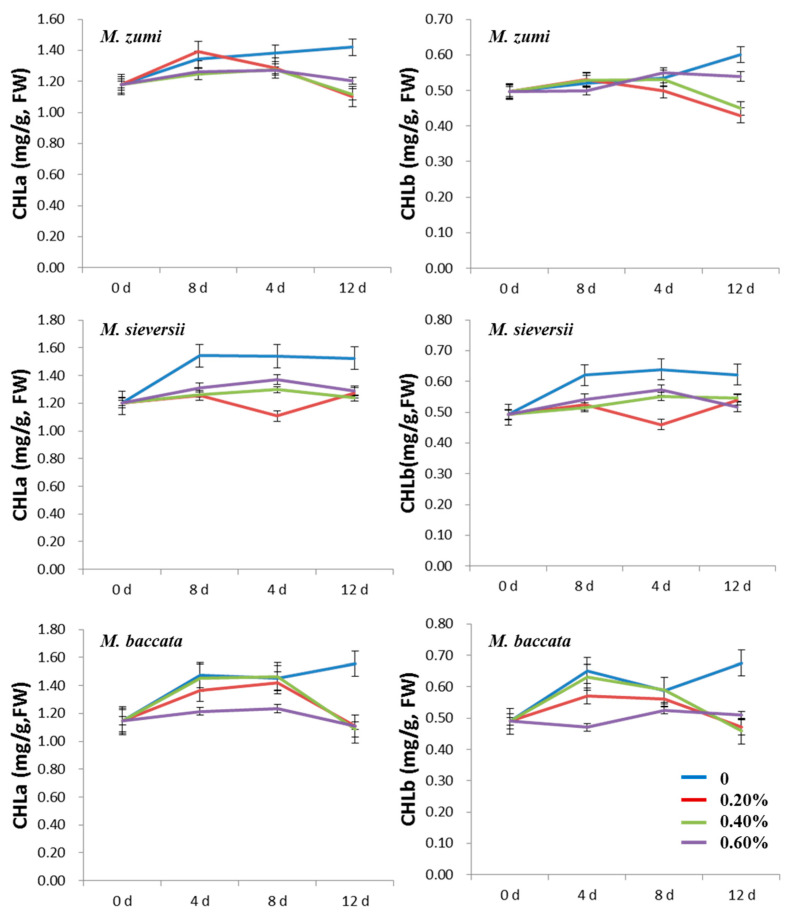
Change curve of leaf CHLa and CHLb contents in the three *Malus* species under four levels of NaCl treatments.

Increasing the salinity concentration reduced the measured values of traits related to photosynthesis. The CHLa and CHLb content in the three *Malus* species decreased with prolongation of the NaCl treatment (Figure 4; Table S1). The values of all treatments were lower than those of the control in *M. baccata*, and there was no significant difference between treatments and control for *M. zumi* except on the twelfth day after NaCl treatment. Moreover, the CHLa and CHLb contents were lower in the 0.60% NaCl treatment groups than in the control for *M. sieversii*. In addition, after 12 days of NaCl treatment, the CHLa and CHLb contents were obviously higher in the control than in the treatments used for the three species.

### 3.2. Dynamic Effects on Physiological Parameters of Malus Plant Roots under Salinity Stress

**Figure 5:**
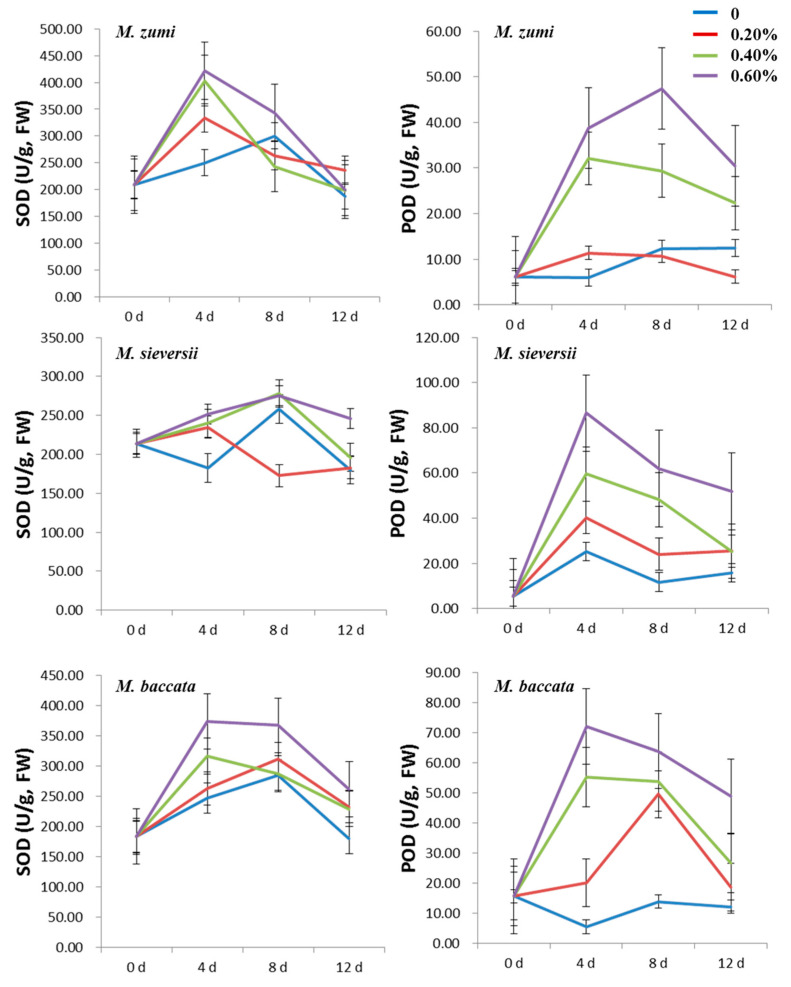
Change curve of root SOD and POD activities in the three *Malus* species under four levels of NaCl treatments.

**Figure 6:**
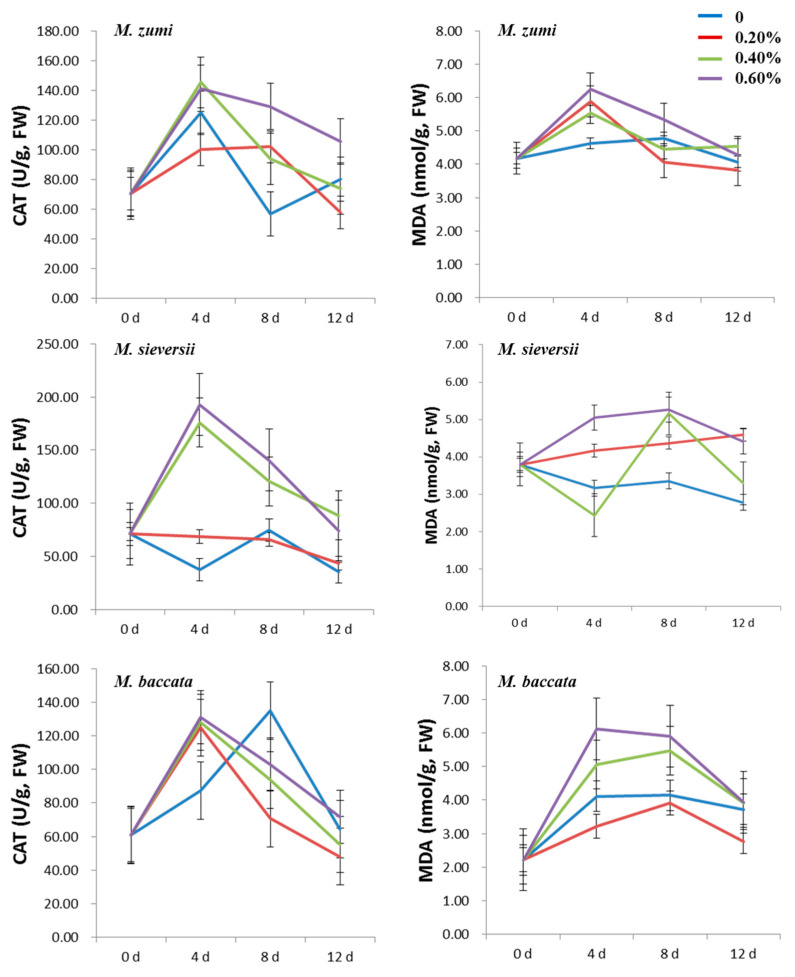
Change curve of root CAT activity and MDA in the three *Malus* species under four levels of NaCl treatments.

During the treatment periods, the SOD, POD, and CAT activities exhibited a similar trend in the roots as in leaves for the three *Malus* species (Figure 5 and Figure 6; Table S2). With the increase in the NaCl concentration, the SOD, POD, and CAT activities were higher in the 0.40% and 0.60% NaCl treatment groups than in the 0.20% NaCl treatment group and control. At the time of peak of these enzyme activities in the 0.60% NaCl treatment group, the SOD activity increased by 6.70%, 51.35%, and 68.23% for *M. sieversii*, *M. baccata*, and *M. zumi*, respectively, compared with the control treatment; the POD activity increased by 243.32%, 1206.89%, and 285.30%, respectively; and the CAT activity increased by 409.05%, 49.98%, and 13.17%, respectively. After 12 days of treatment, there was little difference between treatments and the control for SOD in *M. zumi* or CAT in *M. baccata*, but the activity of POD remained higher than the control in the three species.

The MDA content exhibited a similar trend in the leaves as in the roots for the three *Malus* species (Figure 6; Table S2). With the increasing NaCl concentration, the MDA content was higher in the 0.40% and 0.60% NaCl treatment groups than in the 0.20% NaCl treatment group and in the control for *M. sieversii* and *M. baccata*, but there was no significant difference among treatments for *M. zumi*. At the time of peak in the 0.60% NaCl treatment group, the MDA content in *M. sieversii*, *M. baccata*, and *M. zumi* increased by 20.59%, 48.54%, and 34.77%, respectively, compared with the control. After 12 days of NaCl treatments, the MDA content tended to normal levels in *M. zumi* but were still higher in the treatments than in the control in *M. sieversii* and *M. baccata*.

**Figure 7:**
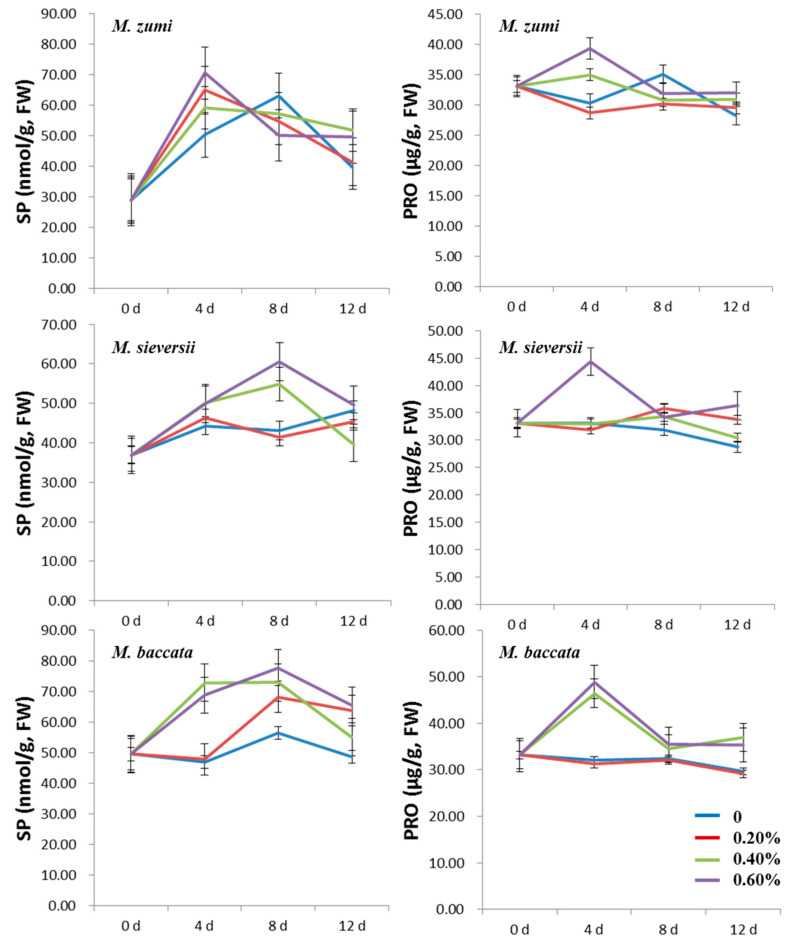
Change curve of root SP and PRO contents in the three *Malus* species under four levels of NaCl treatments.

The SP and PRO contents increased at first and then decreased in the three *Malus* species (Figure 7; Table S2). The SP and PRO contents were higher in the 0.40% and 0.60% NaCl treatment groups than in the 0.20% NaCl treatment group and the control. There was a difference in the peak time of SP; i.e., it was on the fourth day after NaCl treatments for *M. zumi* but on the eighth day after NaCl treatment for *M. sieversii* and *M. baccata*. With the increasing NaCl concentration, the SP and PRO contents were lower in the 0.20% NaCl treatment group and the control than in the 0.40% and 0.60% groups for the three *Malus* species. Considering the peak SP and PRO contents in the 0.60% NaCl treatment group, the SP content increased by 40.22%, 46.32%, and 40.17% for *M. sieversii*, *M. baccata*, and *M. zumi*, respectively; and the PRO content increased by 34.03%, 52.26%, and 29.75%, respectively, compared with the control treatment. After 12 days of treatment, there was little difference between the treatments and control for SP and PRO in *M. zumi* and *M. sieversii*, while they remained higher in treatments than in the control in *M. baccata*.

### 3.3. Comparing the Effects on Physiological Parameters of Roots and Leaves of Malus Plants under Salinity Stress

The mean values of the physiological parameters were compared between the treatments and control (Tables S1 and S2), and significant differences at the 0.05 level were determined. The results showed that the SOD and POD activities were higher in the roots than in the leaves and that the MDA, SP, and PRO contents were higher in the leaves than in the roots, while there was no significant difference in CAT activity between leaves and roots. Under salt stress, the increase in SOD, POD, CAT, and MDA was higher in the roots than in the leaves, whereas the increases in SP and PRO were slightly higher in the leaves than in the roots.

**Figure 8:**
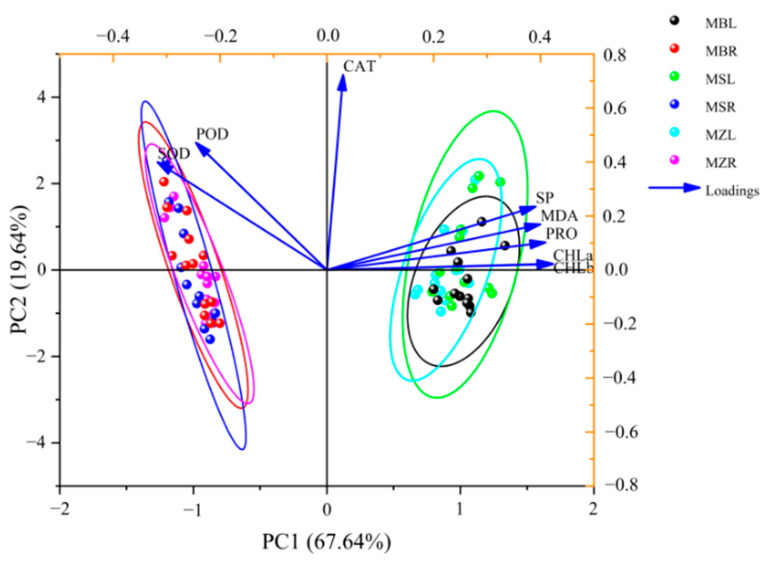
PCA of physiological parameters in leaves and roots for the three *Malus* species. Loading and scores plot of the first two principal components of the principal component analysis model. The left and bottom coordinates were the loading scores of the first two principle components, and the top and right coordinates were the scores of all the physiological parameters in the first two principle components. MZL—leaves of *M. zumi*; MSL—leaves of *M. sieversii*; MBL—leaves of *M. baccata*; MZR—roots of *M. zumi;* MSR—roots of *M. sieversii*; MBR—roots of *M. baccata*.

The PCA, which included all physiological parameters from both the leaves and roots, showed that there was a significant difference between these tissues (Figure 8). Principal component 1 (PC1) and principal component 2 (PC2) could explain 67.64% and 19.64% of the variation, respectively, so the first two principle components could thus explain 87.28% of the variation. Leaves and roots could be distinguished by PC1, which mainly presented CHLa and CHLb, and were positively correlated. Roots were separated from leaves mainly due to a lack of CHLa and CHLb contents, and most of the root physiological parameters were found on the left of the PCA plot. This indicated that the roots had higher SOD and POD activities because they were also found on the left. Thus, SOD and POD were other factors that distinguished roots from leaves.

A PCA using the physiological parameters of the leaves was performed (Figure S1). PC1 and PC2 could explain 36.52% and 23.81% of the variation, respectively, so the first two principle components could thus explain 60.33% of the variation. *M. baccata* could be distinguished from other *Malus* species by PC2, which mainly presented CHLa and CHLb and had lower CHLa and CHLb contents. The other two species could not be distinguished by the PCA of the leaves. A PCA using the physiological parameters of the roots was applied (Figure S2). PC1 and PC2 could explain 59.19% and 14.75% of the variation, respectively, so the first two principle components could thus explain 73.94% of the variation, which was more than in the leaves. *M. zumi* and *M. sieversii* could be distinguished by PC2, which mainly presented POD, PRO, and CAT; *M. sieversii* had higher values for these parameters, while *M. baccata* could not be distinguished from the other two *Malus* species based on the comparison of roots.

## 4. Discussion

Salt ions cause little damage to the cell membranes of highly resistant varieties, and the MDA production is reduced. For the medium-resistance varieties, MDA causes damage to membranes via lipid peroxidation, which leads to the formation of ROS; however, they are able to protect cells from further oxidative damage via their own enzymatic defense systems [35]. The results of this study showed that *M. zumi* and *M. sieversii* had a lower MDA content than *M. baccata* in the NaCl treatment groups. This was possibly due to the higher SOD and CAT activities of *M. zumi* and *M. sieversii*. Moreover, the SOD, POD, and CAT activities were the highest in both the leaves and roots of *M. sieversii* in the control. In addition, *M. baccata* had the lowest SOD and CAT activities in the roots and the lowest POD activity in the leaves. The formation and elimination of ROS were in dynamic equilibrium under normal conditions in the plants. The SOD, POD, and CAT activities exhibited the ability to resist adversity without stress [36,37,38]. *M. zumi* is highly resistant to salt stress, but the enzyme activity in this species was not the highest. Aside from enzymatic antioxidants, non-enzymatic antioxidants also helped to scavenge these indigenously generated ROS [39]. The present results showed that *M. zumi* and *M. sieversii* had a higher PRO content in leaves of the control and that there was no significant difference in the root PRO content of the three species. It was interesting that *M. baccata* had the higher SP content in roots. The high content of osmotic substances in roots helped the plants to cope with water absorption disorders caused by salt stress. We therefore concluded that both enzymatic and non-enzymatic antioxidants played a major role in determining the medium resistance of *M. sieversii* due to its high activity of CAT in the leaves and roots and the content of SP in the leaves. We speculated that there were other endogenous substances that could bear primary responsibility for the high salt resistance of *M. zumi* due to its low contents of SP and PRO. Hannachi et al. [40] confirmed that salicylic acid could be involved in salt-stress tolerance; i.e., it was associated with the efficient antioxidant defense system for scavenging ROS. Various salinized plants showed high values of total soluble sugars and total free amino acids [41,42].

Salt damage was mitigated if a plant developed a series of responses to alleviate the associated stress [43,44,45]. In the present study, the POD, SOD, and CAT activities in the leaves and roots of *M. zumi*, *M. sieversii*, and *M. baccata* increased after NaCl treatments, reaching a peak on the fourth day in most cases. The SOD, POD, and CAT activities increased in order to rapidly scavenge ROS under salt stress [46]. The increasing activities of SOD, POD, and CAT in the first four days after treatments showed that these enzymes were the main substances that were scavenging ROS, while the enzymes that played a major role in the three species differed. In the 0.60% NaCl treatment groups, POD in *M. baccata* increased the most in the both leaves and roots after 4 days of NaCl treatments compared with the control; moreover, the SOD and CAT in *M. sieversii* increased the most in the leaves, and the SOD in *M. zumi* increased the most in the roots. Regarding osmotic regulation, *M. baccata* exhibited a significant increase in the proportions of SP and PRO in the roots. This indicated that *M. baccata* was sensitive to salt stress, and thus higher levels of SOD, POD, and CAT activities were needed to overcome the salt injury. The MDA content in *M. baccata* remained high after 12 days of treatment, by which point the enzyme activities and MDA content in *M. zumi* had returned to a normal level. ROS were continuously produced under salt stress, but the damage could be mitigated if they were eliminated in time [43]. It was demonstrated that *M. zumi* had a stronger recovery capability than *M. baccata*.

The SOD, POD, and CAT activities and the MDA, SP, and PRO contents increased with the increase in the NaCl concentration. Moreover, there was a greater accumulation of MDA for *M. baccata* in the different NaCl treatment groups over the same period. When comparing the different NaCl treatments, there was an obvious increase in the leaves of SOD and CAT for *M. sieversii* and in POD and PRO for *M. zumi* and *M. baccata*. In addition, SOD for *M. zumi*, POD and CAT for *M. sieversii* and *M. baccata*, and SP and PRO for *M. baccata* exhibited a significant increase in the roots. In a previous study on Chinese cabbage, the MDA content was shown to continually increase with the increase in the NaCl concentration [47]. In addition, sensitive cultivars were shown to accumulate more MDA in eggplant seedlings [48], and reduced MDA accumulation was a reflection of improved growth performance under salinity stress [49]. Various studies concluded that the level of PRO content should not be used as an indicator of salt resistance [44,45,50,51,52], while other studies reported that its increase under biological or abiotic stress was a type of victimization symptom. NaCl had little effect on the PRO content in salt-tolerant *Malus* plants, whereas the content in salt-sensitive species continued to increase significantly under a high salt concentration [53,54,55]. In our study, *M. baccata* suffered a more serious injury than the other species under the same NaCl treatments. This was mainly due to the lower CAT and SOD activities in both the roots and leaves under these treatments and to the lower SP content in the roots, although the activities of the aforementioned enzymes were high in the control. In addition, PRO accumulation occurred in both the leaves and roots of all three species when subjected to NaCl treatments, which supported the hypothesis that PRO accumulation was a symptom of salt damage and could be an indicator of resistance to salt in the *Malus* species.

Chlorophyll content is one of the most important indexes of photosynthesis. In this study, the CHLa and CHLb contents were slightly lower in the NaCl treatments than in the control for *M. zumi* and *M. sieversii*, while a significant difference between all NaCl treatments and the control was observed in *M. baccata*. Chlorophyll was suppressed with an increase in salinity [56,57]. Other reports have shown that chlorophyll contents were higher under salinity stress conditions [58]. The results of this study showed that salt stress may inhibit photosynthesis in *M. baccata*.

The main substances for scavenging ROS are different under salt stress in different plants. Responses to salt stress even differed in different tissues within the same plant. The SOD and POD activities were higher in the roots than in the leaves of the three *Malus* species. The SP and PRO contents were higher in the leaves than in the roots, but no significant differences in CAT were observed. Salt damage was directly harmful to plant roots, but the increasing proportion of MDA in the leaves was higher than in the roots in the three species for almost all periods of treatment. Both the leaves and roots responded to salt stress in plants. In a previous study, it was noted that root growth was positively correlated with aboveground growth and that the changes in physiological parameters of the leaves and roots varied for different *Malus* species [59]. We obtained similar results, which indicated that the roots were more tolerant to salt than the leaves. Moreover, the roots were shown to play a key role in eliminating ROS under salt stress in *Malus* plants, and the degree of damage under salt treatment was more serious to the leaves than the roots. Based on the PCA, we concluded that SOD and POD activities and CHLa and CHLb contents were the main factors that differentiated between the roots and leaves of *Malus* species under salt stress. Moreover, *M. zumi* and *M. baccata* were distinguished by POD, PRO, and CAT in the roots or MDA, SOD, and SP in the roots. *M. baccata* could be distinguished from *M. zumi* and *M. sieversii* by the CHLa, CHLb, and PRO contents in leaves.

## 5. Conclusions

The activity and content of the measured physiological parameters were higher in the 0.40% and 0.60% NaCl treatment groups than in the 0.20% group and the control. The SOD and POD activities were higher in the roots than in the leaves; while the MDA, SP, and PRO contents were higher in the leaves than in the roots. The resistance to salt stress of *M. zumi* was mainly due to the high SOD and POD activities under salt stress, while there were other substances that also may have played a major role in the salt stress response. Osmotic regulation was shown to play a greater role in the response to salt stress than enzymatic antioxidants in *M. baccata*, and both enzymatic antioxidants and osmotic regulation made a significant contribution to salt resistance in *M. sieversii*.

## Data Availability

All data generated or analyzed during this study are included in the published article.

## Supplementary Materials

The following supporting information can be downloaded at: https://www.mdpi.com/article/10.3390/life12111929/s1, Table S1: ANOVA analysis of physiological parameters in leaves for the three *Malus* species; Table S2: ANOVA analysis of physiological parameters in roots for the three *Malus* species; Figure S1: PCA of physiological parameters in leaves for the three *Malus* species; Figure S2: PCA of physiological parameters in roots for the three *Malus* species.
