# Supplementary material for: Effects of Salt Stress on the Antioxidant Activity and Malondialdehyde, Solution Protein, Proline, and Chlorophyll Contents of Three Malus Species
Source: Life (Basel). 2022 Nov 18;12(11):1929. doi: 10.3390/life12111929 (PMC9696785; doi:10.3390/life12111929)
Supplement: Supplementary file 1 [file life-12-01929-s001.zip › Figures S1 and S2.pdf]

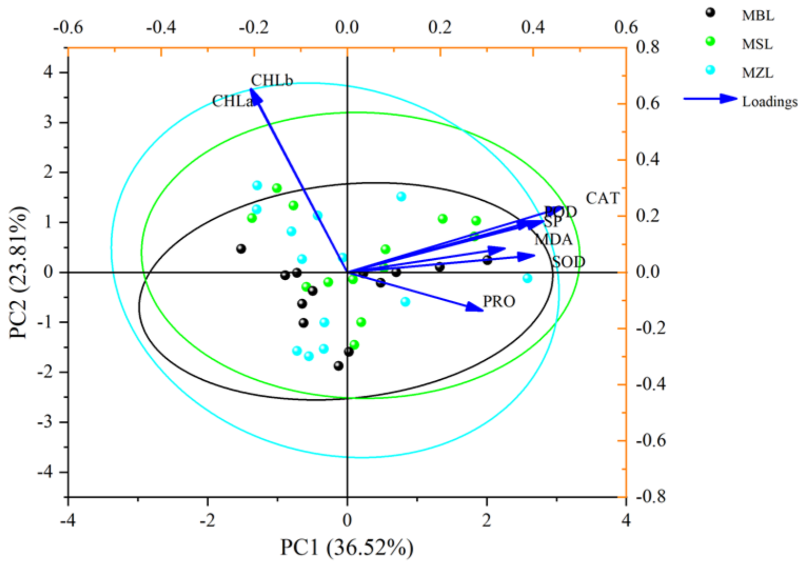

Figure S1. PCA of physiological parameters in leaves for the three *Malus* species. Loading and scores plot of the first two principal components of the principal component analysis model. The left and bottom coordinates represented the loading scores of the first two principle components, and the top and right coordinates represented the score of the all physiological parameters in the first two principle components (MZL—leaves of *M.zumi*; MSL—leaves of *M.sieversii*; MBL—leaves of *M.baccata*).

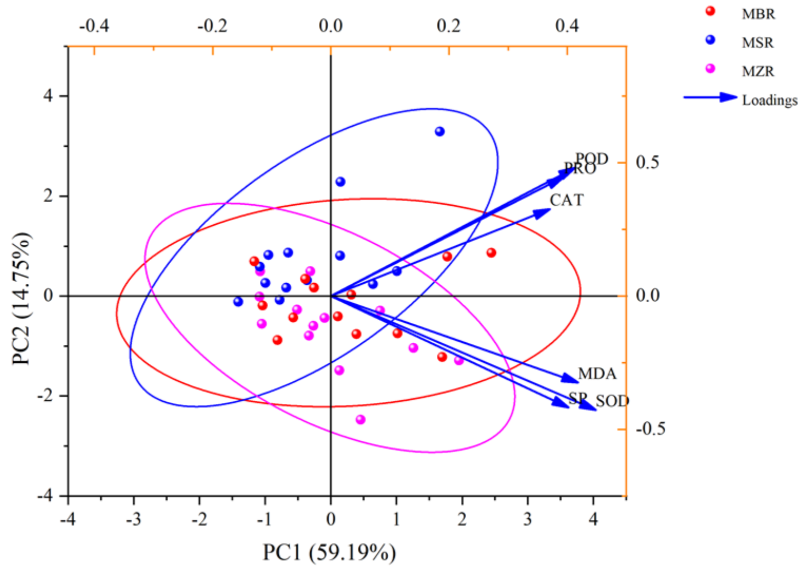

Figure S2. PCA of physiological parameters in roots for the three *Malus* species. Loading and scores plot of the first two principal components of the principal component analysis model. The left and bottom coordinates represented the loading scores of the first two principle components, and the top and right coordinates represented the score of the all physiological parameters in the first two principle components (MZR—roots of *M. zumi;* MSR—roots of *M. sieversii*; MBR—roots of *M. baccata*).
